# Ketone monoester attenuates declines in cognitive performance and oxygen saturation during acute severe hypoxic exposure under resting conditions

**DOI:** 10.1113/EP091794

**Published:** 2024-08-27

**Authors:** Tyler S. McClure, Jeffrey Phillips, Andrew P. Koutnik, Kody Coleman, Ed Chappe, Gary R. Cutter, Brendan Egan, Todd Norell, Brianna J. Stubbs, Marcas M. Bamman, Dawn Kernagis

**Affiliations:** ^1^ School of Health and Human Performance Dublin City University Dublin Ireland; ^2^ Healthspan, Resilience and Performance Research Florida Institute for Human and Machine Cognition Pensacola Florida USA; ^3^ Sansum Diabetes Research Institute Santa Barbara California USA; ^4^ Buck Institute for Research on Aging Novato California USA; ^5^ Department of Neurosurgery University of North Carolina Chapel Hill North Carolina USA

**Keywords:** β‐hydroxybutyrate, code substitution, cognitive resilience, exogenous ketosis

## Abstract

Exogenous ketone supplements are a potential augmentation strategy for cognitive resilience during acute hypoxic exposure due to their capacity to attenuate the decline in oxygen (O_2_) availability, and by providing an alternative substrate for cerebral metabolism. Utilizing a single‐blind randomized crossover design, 16 male military personnel (age, 25.3 ± 2.4 year, body mass, 86.2 ± 9.3 kg) performed tests of cognitive performance at rest in three environments: room air (baseline), normoxia (20 min; 0 m; 20.9% O_2_) and hypoxia (20 min; 6096 m, 9.7% O_2_) using a reduced O_2_ breathing device (ROBD). (*R*)‐3‐Hydroxybutyl (*R*)‐3‐hydroxybutyrate (R‐BD R‐βHB) ketone monoester (KME; 650 mg/kg, split dose given at 30 min prior to each exposure) or taste‐matched placebo (PLA) was ingested prior to normoxia and hypoxic exposure. Blood R‐βHB and glucose concentrations, cognitive performance and O_2_ saturation ($S_{pO_{2}}$) were collected throughout. KME ingestion increased blood R‐βHB concentration, which was rapid and sustained (>4 mM 30 min post; *P *< 0.001) and accompanied by lower blood glucose concentration (∼20 mg/dL; *P *< 0.01) compared to PLA. Declines in cognitive performance during hypoxic exposure, assessed as cognitive efficiency during a Defense Automated Neurobehavioral Assessment (DANA) code substitution task, were attenuated with KME leading to 6.8 (95% CL: 1.0, 12.6) more correct responses per minute compared to PLA (*P* = 0.018). The decline in $S_{pO_{2}}$ during hypoxic exposure was attenuated (6.40% $S_{pO_{2}}$; 95% CL: 0.04, 12.75; *P* = 0.049) in KME compared to PLA (KME, 76.8 ± 6.4% $S_{pO_{2}}$; PLA, 70.4 ± 7.4% $S_{pO_{2}}$). Acute ingestion of KME attenuated the decline in cognitive performance during acute severe hypoxic exposure, which coincided with attenuation of declines in O_2_ saturation.

## Abstract (highlights)

- **What is the central question of this study?**
  Can exogenous ketosis act as a countermeasure to declines in blood oxygen saturation and cognitive performance during acute severe hypoxic exposure at rest?
- **What is the main finding and its importance?**
  Acute exogenous ketosis via ingestion of a drink containing the (*R*)‐3‐hydroxybutyl (*R*)‐3‐hydroxybutyrate ketone monoester prior to acute severe hypoxic exposure attenuated hypoxia‐induced declines in blood oxygen saturation and cognitive performance at rest.

## 1 INTRODUCTION

Hypoxia can occur during altitude exposure or pathologically through cardiovascular disease, asthma, chronic obstructive pulmonary disease (COPD), or respiratory viral infections. The associated reduction in oxygen (O_2_) availability diminishes mitochondrial respiratory rate and increases oxidative stress and inflammation (Pasiakos et al., 2021), which has a negative impact on multiple organ and tissue systems, including the brain and musculoskeletal system amongst others (Chaillou, 2018; Goodall et al., 2014; Michiels, 2004). Acute hypoxic exposure can lead to a decline in circulating haemoglobin O_2_ saturation ($S_{pO_{2}}$), affecting cerebral metabolism, which can result in impaired cognitive function (Williams et al., 2019). Moderate‐to‐severe hypoxic exposure has been linked to acute disruptions in working memory (Legg et al., 2016; Malle et al., 2013), cognitive flexibility (Asmaro et al., 2013; Turner et al., 2015), attention (Asmaro et al., 2013; Stepanek et al., 2014; Turner et al., 2015), executive function (Turner et al., 2015) and auditory processing (Beer et al., 2017).

Countermeasures that attenuate or eliminate the effects of hypoxia may have far‐reaching implications for those affected by, or at risk for, hypoxia. Attenuating declines in circulating and tissue O_2_ concentrations is a promising target to alleviate the symptoms of acute hypoxia and mitigate negative impacts of long‐term hypoxia. For example, descending to a lower altitude and increasing breathing rate through hyperventilation during environmental hypoxia can alleviate acute altitude‐related hypoxia symptoms (Shaw et al., 2021). Daily O_2_ therapy has been demonstrated to improve cognition and mortality rates in COPD patients with chronic pathological hypoxia (Karamanli et al., 2015; Pavlov et al., 2018). Thus, whether hypoxia symptoms are acute or chronic, alleviating them is most effectively achieved by increasing oxygen availability. Furthermore, the use of the carbonic anhydrase inhibitor acetazolamide, which induces mild acidosis and stimulates respiratory rate, has been demonstrated to raise O_2_ saturation and attenuate acute mountain sickness (AMS) symptoms (Wang et al., 2015). Although acetazolamide is the standard of care for treating AMS, and has been shown to decrease the incidence of AMS at high altitude, studies have also demonstrated negative effects of carbon anhydrase inhibitors on sub‐maximal and maximal physical performance (Posch et al., 2018) and learning, memory and attention (Sun & Alkon, 2002; Wang et al., 2013). Treatments that are effective at low‐to‐moderate altitudes are also often ineffective in more severe hypoxic environments (Robach et al., 2008). These limitations demonstrate a clear gap for special populations, such as military aviation pilots, mountain warfare operators and rescue operation personnel, amongst others who must perform cognitively demanding missions at altitudes up to and on occasion exceeding 6096 m (Shaw et al., 2021), especially those requiring rapid ascents. Even minor degradation of these processes can meaningfully decrease the likelihood of success under these extreme conditions and can have severe consequences. Given these factors, there is a pressing need to find more effective and practical countermeasures for hypoxic exposure.

A ketogenic diet (KD), hallmarked by an elevation in circulating ketone bodies (KB), referred to as ketosis, has demonstrated the potential to function as a countermeasure for hypoxic conditions in preclinical models (Brownlow et al., 2017; Gom et al., 2021). However, the KD requires a multiweek adaptation timeline and has the potential for reduced compliance over time (Burke, 2021). Acute ingestion of KBs to induce ketosis through exogenous ketone supplements may be a more practical option as they have been demonstrated to rapidly elevate blood *R*‐β‐hydroxybutyrate (*R*‐βHB) without requiring lifestyle or dietary manipulations (Evans et al., 2022; Poff et al., 2020). Our laboratory recently demonstrated the potential ability for the (*R*)‐3‐hydroxybutyl (*R*)‐3‐hydroxybutyrate (R‐BD R‐βHB) ketone monoester (KME) to mitigate the negative effects on cognitive performance of acute moderate hypoxic exposure (5029 m) (Coleman et al., 2021). However, military operators are often required to maintain high levels of cognition across diverse tasks when rapidly deployed into severely hypoxic environments. Thus, the aim of the present study was to investigate the impact of acute ingestion of KME on cognitive resilience during acute severe hypoxia (simulated 6096 m altitude; 9.7% O_2_), coupled with assessments of blood oxygen saturation and metabolic, cardiac and autonomic function. We hypothesized that the acute ingestion of R‐BD R‐βHB KME would attenuate the declines in $S_{pO_{2}}$ and cognitive performance caused by acute severe hypoxic exposure.

## 2 METHODS

### 2.1 Ethical approval

Each participant provided written informed consent to participate after written and verbal explanations of the procedures in accordance with the protocol approved by the Florida Institute for Human and Machine Cognition Institutional Review Board (IRB‐2019‐005; IRB‐2021‐0011) and Office of Human Research Oversight, U.S. Army Medical Research and Development Command, and followed DoD Instruction 3216.02, ‘Protection of Human Subjects and Adherence to Ethical Standards in DoD‐Conducted and Supported Research’ (E02621.1b). The study conformed to the standards set by the *Declaration of Helsinki*, except for registration in a trials database.

### 2.2 Participants

Twenty‐three male military aviation students aged 18–35 years stationed at Naval Air Station Pensacola, FL, USA were recruited to participate in the study (age, 25.3 ± 2.4 years, body mass, 86.2 ± 9.3 kg). Each participant provided written informed consent to participate after written and verbal explanations of the procedures in accordance with the protocol approved by the Florida Institute for Human and Machine Cognition Institutional Review Board. Inclusion criteria were: (1) 18–35 years of age and (2) regular consumption of a typical American diet (Snetselaar et al., 2021). Exclusion criteria were: (1) history of smoking, (2) metabolic or cardiovascular disease, (3) orthopaedic, musculoskeletal, neurological, psychiatric disorder, and/or any other medical condition that prohibited exercise, (4) habitual prescription medications, (5) exogenous ketone supplements or following a specialized dietary pattern, for example, low‐carbohydrate or KD (Volek et al., 2024), (6) ergogenic aids within 1 month of study participation, and (7) recent (within last 30 days) altitude/hypoxic exposure. Participants were instructed to refrain from caffeine for 12 h, alcohol consumption and training for 24 h, and arrive in an overnight fasted state before each experimental trial. Participants were recreationally active, and instructed to maintain their usual exercise training frequency and volume throughout the study intervention. During the study, *n* = 7 participants did not complete the protocol: three volitionally withdrew and four were administratively withdrawn for safety reasons (during severe hypoxic exposure, two dropped below pre‐determined $S_{pO_{2}}$ safety threshold ($S_{pO_{2}}$ ≤ 58%) and two had attenuated responsiveness verified via verbal communication and eye responsiveness). All data from these participants were excluded from the final analysis. Data analyses were thus performed based on the *n* = 16 participants who completed both experimental visits.

### 2.3 Experimental design

**FIGURE 1:**
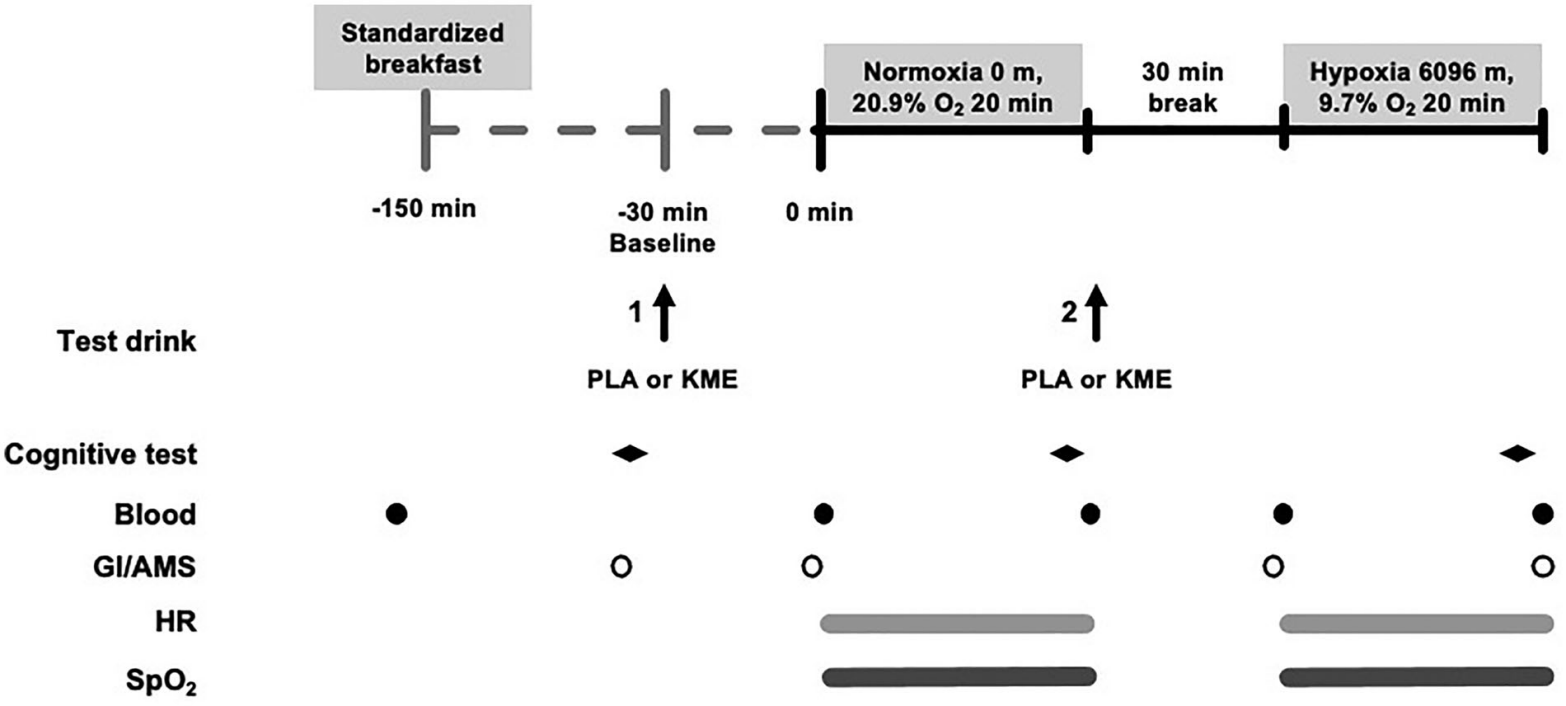
Schematic illustration of the experimental design. This study investigated whether acute ingestion of R‐BD R‐βHB KME impacts cognitive performance and $S_{pO_{2}}$ at rest after exposure to rapid‐onset acute severe hypoxia.

A single‐blind, placebo‐controlled, randomized crossover design was used and participants visited the laboratory on three separate occasions over a 10‐ to 16‐day period, comprising one familiarization and two main experimental visits with a ≥48 h wash‐out period between experimental visits. During the first visit, participants were familiarized with the protocol, equipment, and all cognitive tests (code substitution task, RightEye oculometric test). The two main experimental trials (visits 2 and 3) consisted of cognitive assessments performed under resting conditions in normoxia followed 30 min later by acute exposure to hypoxia on a reduced oxygen breathing device (ROBD) (Figure 1). Visits 2 and 3 were identical in terms of pre‐test preparation for 24 h (no physical activity and participants instructed to replicate diet) and the experimental trials. The visits differed only in the randomly assigned drink consumed before undergoing cognitive testing during hypoxia, in the form of a volume, colour and flavour‐matched placebo (PLA) or R‐BD R‐βHB KME. Within participants, PLA versus KME was randomized for visits 2 and 3. The primary outcome was cognitive performance with secondary outcomes including blood oxygen saturation, heart rate (HR), AMS symptoms, and circulating βHB and glucose concentrations.

### 2.4 Cognitive assessments

At Baseline, and the end of the sessions of normoxia and hypoxia, participants performed assessments of cognitive performance. Cognitive performance testing at each of these time points consisted of RightEye oculometric measurement followed immediately by the Defense Automated Neurobehavioral Assessment (DANA), and always in that order. A familiarization test was performed during the first laboratory visit to reduce the possibility of a learning effect. All tests were performed in a sound‐insulated room under controlled conditions (i.e., appropriate lighting, as quiet as possible, and isolation from unnecessary stimuli and timing feedback). Participants were instructed to complete the battery as quickly and accurately as possible. Each trial was administered identically.

#### 2.4.1 RightEye oculometric measurement

Participants completed an eye‐tracking cognitive assessment (RightEye, Bethesda, MD, USA) once at baseline and during each experimental trial (Normoxia, Hypoxia) for three total time points in each trial. At each time point, participants were aligned with the RightEye reading device and instructed to read a short prompt and answer Yes or No comprehension questions based on what they had just read. During this time, accuracy, reading rate, blink rate and duration were all recorded for later analysis. Test–re‐test reliability as intra‐class correlation coefficient (ICC) for RightEye has been previously published (horizontal ICC: 0.83–0.87; vertical ICC: 0.89–0.94) (Roberts et al., 2017).

#### 2.4.2 DANA

Participants completed a simultaneous and delayed code substitution task on a self‐directed tablet‐based DANA (AnthroTronix In, Silver Springs, MD, USA) once at baseline and during each exposure (normoxia, hypoxia) for three total time points in each experimental trial. The simultaneous code substitution (CSS) was first, followed by delayed (CSD). For CSS, the participant refers to a code of nine symbol–digit pairs displayed across the upper portion of the screen. A sequence of single symbol–digit pairs is shown below the key, and the participant indicates whether or not the single pair matches the code by pressing Yes or No. For CSD, the nine symbol–digit pairs are not displayed on the screen. However, the participant is still shown a sequence of single symbol–digit pairs and is asked to recall from memory if these are the correct pairs from the CSS session that just occurred, as the symbol–digit pairs change at each time point. During this time, accuracy, reaction time (RT), correct reaction time (CRT) and cognitive efficiency (CE, correct responses per minute) were recorded for later analysis. Test–re‐test reliability as ICC for DANA has been previously published (CSS: 0.88; CSD: 0.54) (Coleman et al., 2021; Lathan et al., 2013).

### 2.5 Experimental trials

After consenting to participate, participants underwent a familiarization visit involving practice of the cognitive tests and familiarization with the equipment. Dietary intake data for (24‐h recall) were collected and analysed using the automated self‐administered 24‐h (ASA24) Dietary Assessment Tool, version 2021 (Subar et al., 2012), developed by the National Cancer Institute (Bethesda, MD, USA), to ensure subjects were not currently consuming a KD.

Participants arrived in the morning (07.00–08.00 h) in a fasted state, completed a behavioural compliance questionnaire, provided a urine sample for urine specific gravity (USG) and were weighed. After a baseline blood sample was collected, participants were fed a standardized breakfast of a bagel (kcal: 260; fat (F): 3 g; carbohydrate (C): 48 g; protein (P): 11 g) with a single serving of peanut butter (kcal: 250; F: 21 g; C: 11 g; P: 9 g). After consumption, participants rested for approximately 2 h before ingesting KME (Pure (∆)G Ketone Ester HVMN Ketone; HVMN, Inc., San Francisco, CA, USA) or PLA. During the rest period, participants completed a baseline cognitive assessment (RightEye, DANA). Participants were given 5 min at each time point to consume two separate drinks of KME or PLA each visit. The first drink was administered 30 min prior to beginning the normoxia session. The second drink was administered after completing the normoxia session and 30 min prior to the hypoxia session. Based on our previous work, this dosing schedule maintains blood R‐βHB over 4 mM for both normoxia and hypoxic exposure (Coleman et al., 2021). Doses of KME were based on body weight (drink 1: 500 mg/kg; drink 2: 150 mg/kg) while PLA was a taste, volume and colour‐matched non‐caloric drink containing 2.6 mL of matched flavoring (HVMN Inc.), 0.87 mL of a bitter flavour stock (Bitrex, Edinburgh, UK), 0.13 mL of artificial sweetener (Truvia, San Diego, CA, US), and 26 mL of water. This formulation was piloted to ensure effective blinding. Immediately after ingesting the study drink participants consumed approximately 20 mL of a calorie‐free liquid water enhancer (Mio, Kraft Heinz, Chicago, IL, USA) to remove any lingering flavour. To ensure the participants and the researcher conducting the cognitive testing were blinded to the condition, the study drinks were prepared by the researcher who collected blood samples and served it in an unmarked paper cup. The participants and researcher assigned to collect cognitive data were blinded to the assigned condition. Only the researcher completing the blood sampling for ketone and glucose concentrations was aware of the participant's blinded drink assignment due to inability to blind blood ketone concentrations. KME and PLA data sets were coded during all data entry, QC and statistical analyses.

Thirty minutes after the consumption of drink 1, a second blood sample was taken, after which the participants were attached to the ROBD and began the normoxia session (20 min at 20.9% O_2_). Wearing the ROBD during the normoxia testing reduced any possible confounding influence of additional work of breathing due to the ROBD or physical distraction of the device. After 5 min of exposure, cognitive testing began (RightEye followed by DANA). Once completed, the ROBD was removed and the second drink was administered followed by a 30 min rest period. A third blood sample was taken prior to beginning the hypoxic session (20 min at 9.7% O_2_) on the ROBD. After 5 min of exposure, the cognitive testing began (RightEye followed by DANA). Once completed, the ROBD was removed from the participant and the blood sample was taken.

HR was measured (V800 polar, Polar, Kempele, Finland) throughout the normoxia and hypoxia sessions. Systemic oxygen saturation ($S_{pO_{2}}$) was measured each second by pulse oximetry with the Nellcor Bedside Respiratory Patient Monitoring System (Covidien, Dublin, Ireland) placed upon the forehead over the supraorbital artery during the normoxia and hypoxia sessions.

### 2.6 Blood sample analysis

Finger capillary blood was analysed for R‐βHB and glucose concentrations using a clinical grade point of care device (Precision Xtra, Abbott Diabetes Care Inc., Almeda, CA, USA). Blood samples were measured before the standardized breakfast and KME supplementation (Baseline), 30 min after supplementation (Pre‐normoxia), at the end of the normoxia session (Post‐normoxia), immediately before (Pre‐hypoxia), and after the hypoxic exposure (Post‐hypoxia). Fingertip blood samples were collected using a lancet following alcohol cleaning. The first droplet was wiped away with a cotton swab, and the subsequent droplets were used for analysis.

### 2.7 Subjective measures of gastrointestinal symptoms and AMS

At Baseline, Pre‐normoxia, Pre‐hypoxia and Post‐hypoxia, participants were asked to rate on a Likert scale (0–8, 0: no symptoms, 8: unbearable symptoms) gastrointestinal symptoms (heartburn, bloating, nausea, vomiting, intestinal cramps, abdominal pain, flatulence, diarrhoea) as well as symptoms of AMS (dizziness, headache, muscle cramp, urge to urinate).

### 2.8 Statistical analysis

All statistical analyses and graphical representation of data were performed using Prism v9 (GraphPad Software, Boston, MA, USA). Normality of data was assessed with the Shapiro–Wilk normality test, for which all data passed. Data are presented as means ± SD, or mean difference (lower, higher 95% confidence limits of mean) where indicated. A two‐way (Time × Condition) repeated measures ANOVA was used to identify differences if any between the conditions across time. When a Time × Condition interaction effect or a main effect of Condition was observed, *post hoc* pairwise comparisons were performed with Šidák's correction applied and multiplicity‐adjusted *P*‐values are reported. Sphericity was not assumed, and the Greenhouse–Geiser correction was applied to all ANOVA analyses. The threshold for statistical significance was set at *P *≤ 0.05 for all tests.

## 3 RESULTS

### 3.1 Blood concentrations of *R*‐βHB and glucose

**FIGURE 2:**
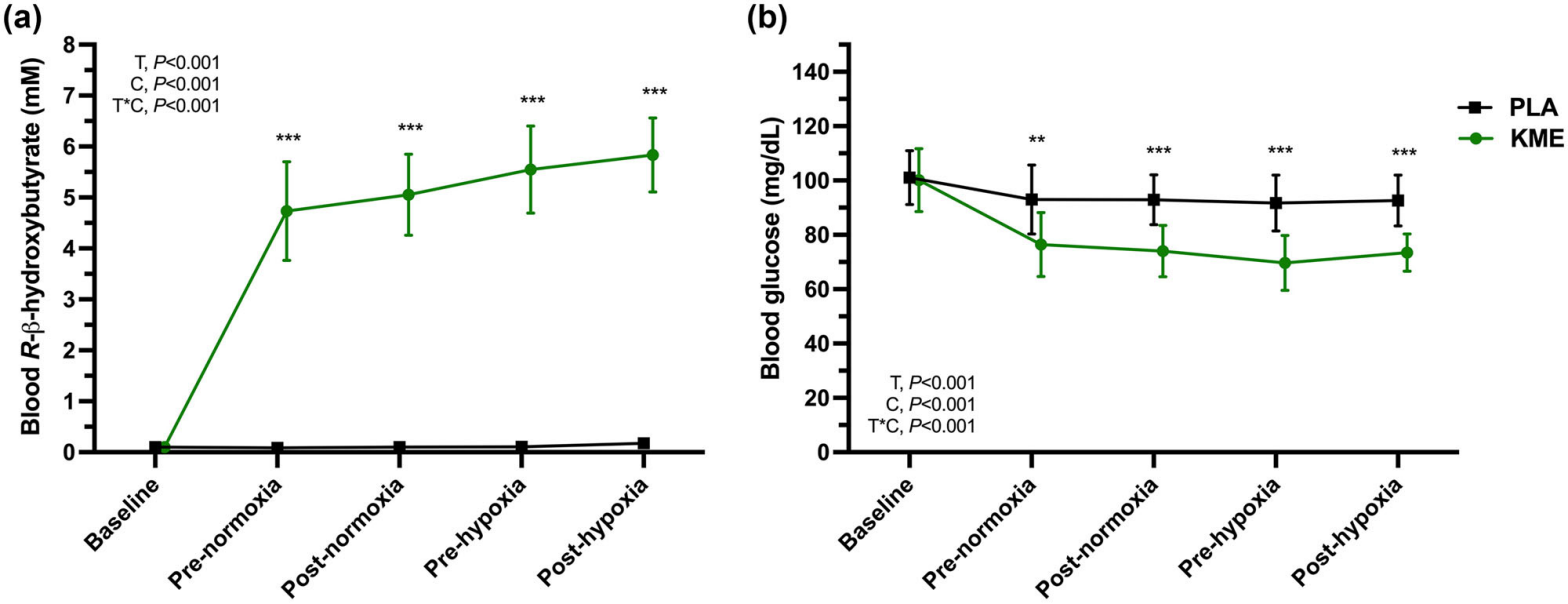
Blood metabolites. Blood *R*‐β‐hydroxybutyrate (a) and glucose (b) at baseline, and before (Pre) and after (Post) normoxia and hypoxia after acute ingestion of an exogenous KME or non‐caloric taste matched PLA. *n* = 16. Data are means ± SD. T, time; C, condition (KME vs. PLA); T*C, time × condition interaction. **P < 0.01 and ***P < 0.001 for KME versus PLA. KME, ketone monoester; PLA, placebo.

For baseline values prior to consuming study drinks, blood R‐βHB and glucose concentration did not differ between the two experimental trial days (Figure 2). KME significantly increased blood R‐βHB 30 min after KME ingestion (4.5 ± 1.0 mM, *P *< 0.001) compared to baseline, and concentration remained elevated for all time points during normoxia and hypoxia compared to PLA (all pairwise comparisons between PLA and KME, *P *< 0.001). Blood glucose concentration was lower (−16.5 mg/dL; 95% CL: −28.9, −4.2; *P* = 0.004) 30 min after ingestion (i.e., Pre‐normoxia) in KME compared to PLA, and remained lower during normoxia and hypoxic exposure by ∼20 mg/dL (all pairwise comparisons between PLA and KME, *P *< 0.01) (Figure 2).

### 3.2 Cognitive performance

#### 3.2.1 DANA

**FIGURE 3:**
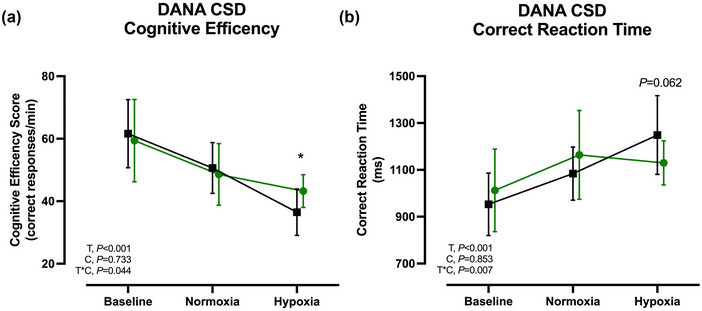
Cognitive performance. DANA CSD cognitive efficiency (a), and DANA CSD correct reaction time (b) at baseline, and before (Pre) and after (Post) normoxia and hypoxia after acute ingestion of an exogenous KME or non‐caloric taste matched PLA. *n* = 16. Data are means ± SD. T, time; C, condition (KME vs. PLA); T*C, time × condition interaction. *P < 0.05 for KME versus PLA. CSD, code substitution delayed; DANA, Defense Automated Neurobehavioral Assessment; KME, ketone monoester; PLA, placebo.

No significant differences between treatments were found at baseline and normoxia for RT, CRT, CE or accuracy for both CSS and CSD (Figure 3; Table 1). During hypoxia, the decline in CE during CSD was significantly attenuated with KME (interaction effect, *P* = 0.044) leading to 6.8 (95% CL: 1.0, 12.6) more correct responses per minute compared to PLA (*P* = 0.018) (Figure 3a).

**TABLE 1:** Cognitive outcomes.

	Time point		
	Baseline	Normoxia	Hypoxia
DANA CSD efficiency (correct response/min)			
PLA	61.5 ± 10.9	50.6 ± 8.1	37.7 ± 9.1
KME	59.4 ± 13.1	47.0 ± 11.6	41.8 ± 7.7*
DANA CSD correct RT (ms)			
PLA	952.8 ± 133.6	1102.9 ± 139.2	1218.1 ± 212.7
KME	1012.4 ± 176.1	1195.3 ± 231.8	1155.2 ± 144.1
DANA CSS efficiency (correct response/min)			
PLA	51.2 ± 7.0	45.0 ± 5.7	40.5 ± 6.3
KME	50.7 ± 9.8	43.7 ± 7.8	41.3 ± 7.0
DANA CSS correct RT (ms)			
PLA	1177.5 ± 172.6	1310.8 ± 172.3	1426.5 ± 220.0
KME	1214.3 ± 234.7	1376.8 ± 210.9	1426.7 ± 230.1
RightEye accuracy (%)			
PLA	87.5 ± 13.4	96.2 ± 5.0	94.3 ± 12.6
KME	86.8 ± 7.9	96.6 ± 4.8	93.7 ± 8.0
RightEye blink rate (blink/min)			
PLA	6.5 ± 4.3	4.9 ± 3.1	5.0 ± 3.8
KME	6.5 ± 6.5	5.4 ± 3.5	4.0 ± 2.7
RightEye blink duration (ms)			
PLA	8.0 ± 5.9	11.3 ± 10.3	12.6 ± 9.1
KME	7.7 ± 6.8	13.8 ± 11.8	18.4 ± 15.2
RightEye reading rate (words/min)			
PLA	179.6 ± 50.8	199.5 ± 62.1	166.4 ± 55.4
KME	157.3 ± 35.5	196 ± 68.7	191.5 ± 66.5

*Note*: Data are presented as means ± SD, *n* = 16. *P < 0.05 for KME versus PLA. Abbreviations: CSD, code substitution delayed; CSS, code substitution simultaneous; DANA, defense automated neurobehavioral assessment; RT, reaction time.

#### 3.2.2 RightEye oculometric assessment

No significant differences occurred between treatments at baseline, normoxia or hypoxia for accuracy, blink duration, blink rate and reading rate (Table 1).

### 3.3 Oxygen saturation and HR

**FIGURE 4:**
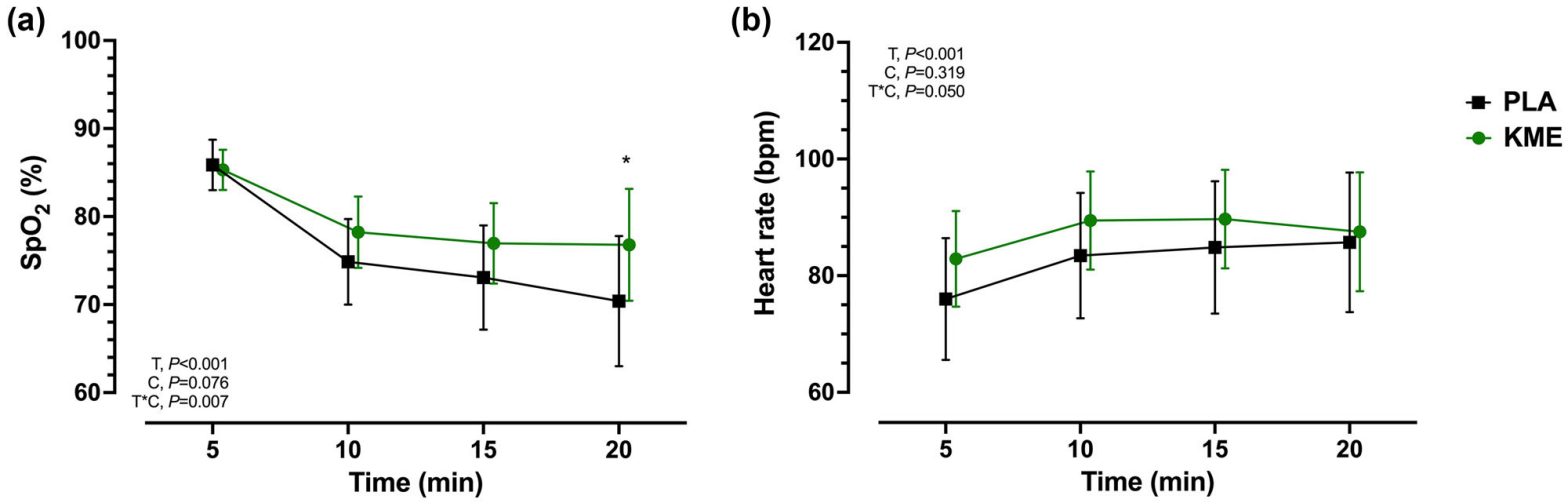
Cardiorespiratory responses. Oxygen saturation (a) and heart rate (b) during hypoxic exposure reported in 5 min intervals after acute ingestion of an exogenous ketone monoester (KME) or non‐caloric taste matched placebo (PLA). *n* = 12. Data are means ± SD. T, time; C, condition (KME vs. PLA); T*C, time × condition interaction. *P < 0.05 for KME versus PLA. KME, ketone monoester; PLA, placebo.

$S_{pO_{2}}$ was not significantly different between treatments during normoxia. KME attenuated the decline in $S_{pO_{2}}$ during the 20 min hypoxic exposure compared to PLA (interaction effect, *P* = 0.007) with the greatest difference occurring during the final 5 min of the hypoxic exposure (6.40% $S_{pO_{2}}$; 95% CL: 0.04, 12.75; *P* = 0.049) (Figure 4a). Resting heart rate was higher in KME during normoxia (KME, 75.0 ± 7.2 bpm; PLA, 66.9 ± 9.8 bpm; *P* = 0.007), and remained directionally elevated (∼5 bpm) during hypoxic exposure, but this difference was not statistically significant (Figure 4b). Of note, for $S_{pO_{2}}$ and HR measurements, technical difficulties during data acquisition resulted in only *n* = 12 in the final analysis of these secondary outcomes.

### 3.4 Gastrointestinal and AMS symptoms

Subjective ratings of heartburn, bloating, nausea, vomiting, intestinal cramps, abdominal pain, flatulence, diarrhoea, dizziness, headache and muscle cramps were not different across treatments in both studies. Therefore, AMS‐related symptoms were negligible at each time point, even with the acute exposure to severe hypoxia employed in this experimental model.

## 4 DISCUSSION

The present study investigated whether acute ingestion of an exogenous ketone supplement in the form of the R‐BD R‐βHB KME could affect cognitive performance in young men acutely exposed to severe hypoxia (simulated 6096 m altitude; 9.7% O_2_). KME rapidly elevated R‐βHB and reduced blood glucose concentrations throughout the study protocol, and an attenuation in the decline in $S_{pO_{2}}$ during hypoxic exposure was observed compared to PLA. These physiological changes induced by KME were associated with attenuation in the decline in cognitive performance in the latter stages of hypoxic exposure.

A divergent effect of cognitive performance was observed between treatment conditions inferring a prophylactic effect against declines in cognitive performance resulting from acute (20 min) severe hypoxic exposure. Specifically, on average 6.8 more correct responses per minute occurred with KME compared to PLA in hypoxia. These results are consistent with observations of cognitive resilience during or following acute stress with KME (Coleman et al., 2021; Evans & Egan, 2018; Murray et al., 2016; Poffé et al., 2023) and at rest in obese subjects (Walsh et al., 2021). However, KME did not confer an improvement in cognitive performance in normoxic conditions suggesting an acute stress was required to observe any effect of KME on cognition. Other supplemental forms of exogenous ketones have observed mixed results. Prins et al. observed cognitive enhancement effects at rest using an exogenous ketone formulation of βHB salts and medium chain triglyceride (MCT) (Prins et al., 2020), whereas another study of βHB salts showed no effect during or after exercise (Waldman et al., 2018). Our results and other literature on exogenous ketones across rest and stress, as well as diverse ketone formulations, suggests a potential interaction between exogenous ketone composition and stress/pathological conditions, as well as divergent mechanisms on cognition across different exogenous ketone formulations (Poff et al., 2020, 2021; Stubbs et al., 2020). The proposed mechanism for ergogenic effects of KME on cognition have been suggested to be via altered substrate availability (Cunnane et al., 2020), elevated BDNF (Walsh et al., 2020), increased cerebral blood flow (Walsh et al., 2021) and/or enhanced brain network stability (Mujica‐Parodi et al., 2020).

The effect of KME to maintain higher cognitive efficiency and faster reaction time during acute severe hypoxic exposure is likely to be elicited through maintaining a higher O_2_ saturation, given that cognitive performance during hypoxic exposure is strongly correlated to $S_{pO_{2}}$ saturation (Ochi et al., 2018) and cerebral metabolic rate (Subudhi et al., 2007). The present study adds to the growing body of literature supporting the ability of acute nutritional ketosis to attenuate the decline in $S_{pO_{2}}$ saturation by approximately 3%–6% during hypoxic exposures at varying simulated altitudes in populations at rest and during exercise (Coleman et al., 2021; Poffe et al., 2021; Prins et al., 2021). We postulate that this enhanced ability to maintain $S_{pO_{2}}$ during hypoxic exposure is mechanistically driven by the acid–base balance response to a rapid influx of KBs. An acute increase in circulating KBs has been previously demonstrated to increase HR and hydrogen ion load, leading to decreases in circulating bicarbonate and ultimately pH (Poffe et al., 2021; Prins et al., 2021). Decreases in pH and O_2_ availability stimulate pulmonary vasoconstriction and lead to hyperventilation (Dunham‐Snary et al., 2017; Leaf & Goldfarb, 2007). This response allows for greater O_2_ uptake and offloading of additional CO_2_ formed from the bicarbonate buffering system (Dunham‐Snary et al., 2017).

Interestingly, the leading treatment of hypoxic exposure is acetazolamide, which also elicits acute metabolic acidosis, although this treatment leads to lower cognitive performance when compared to PLA (Wang et al., 2013). While both compounds induce acute metabolic acidosis to counteract declines in $S_{pO_{2}}$, acute ingestion of KME provides a useable metabolic substrate that has been reported to be more energy‐efficient than glucose, potentially allowing for a greater O_2_ ‘sparing’ effect (Evans et al., 2022). In contrast, acetazolamide uses a carbonic anhydrase inhibitor to shift pH into metabolic acidosis without the benefit of an alternative fuel substrate such as KBs that may help to maintain a high rate of cerebral metabolism (Leaf & Goldfarb, 2007). A recent evaluation of KME during a laboratory‐based simulated altitude ascension combined with exercise suggests greater systemic and muscular O_2_ uptake but no impact on physical performance (Poffe et al., 2021). In contrast, our results in the resting state suggest the $S_{pO_{2}}$ advantage does translate into attenuating a hypoxia‐induced decline in cognitive performance. Thus, future studies should consider addressing whether KME may be a superior treatment strategy to acetazolamide or alternative countermeasures for offsetting hypoxia‐induced performance declines without negatively impacting cognitive and physical performance domains during acute hypoxic exposure.

Hypoxia occurs most prominently during ascension to higher altitudes, but pathological conditions such as asthma, COPD (Sarkar et al., 2017) and respiratory viral infections (Huang et al., 2021) can result in hypoxia and associated symptoms. Interestingly, both diet‐ and exogenous ketone‐induced ketosis have been demonstrated to relieve asthma in pre‐clinical models (Mank et al., 2022). A recent case report demonstrated remarkable improvements in COPD phenotype following diet‐induced ketosis (Norwitz et al., 2021). Hypothetical benefits of KBs have been proposed across multiple domains of respiratory viral infections (Stubbs et al., 2020), some of which were subsequently demonstrated using diet‐induced ketosis in pre‐clinical models of coronavirus (Ryu et al., 2021). Inflammation, oxidative stress and subsequent cognitive decline occur in conditions like asthma and COPD, yet emergent preclinical evidence and case reports (Mank et al., 2022; Norwitz et al., 2021) suggest some utility of KBs in such conditions. The effects are suggested to be a consequence of KBs acting as anti‐inflammatory, antioxidant and epigenetic modifiers (Poff et al., 2020; Stubbs et al., 2020). However, it is unclear whether the acute effects observed herein will translate to chronic pathophysiological conditions and further investigations are required to understand these potential therapeutic applications for KBs.

There are a few limitations in the present study. While we observed differences in oxygen saturation and metabolic, autonomic, and heart rate responses in these healthy, recreationally active male military personnel exposed to acute severe hypoxia with KME administration, whether these results will translate to females or to individuals who are less active is not known. Moreover, this acute hypoxic exposure has a specific context of rapid (<1 h) onset and results may not apply or be relevant in ‘typical’ gradual exposures to altitude over several days, including recommended acclimatization procedures. In fact, this short duration (20 min) exposure did not elicit an increase in AMS‐related symptoms in either condition. The absence of AMS‐related symptoms, even with the acute exposure to severe hypoxia employed in this experimental model, limits the study findings to a very specific situation and context. Follow‐up studies should explore the same research questions around KME for mitigation of declines in cognitive performance in hypoxia, but employ an experimental model wherein AMS symptoms are prominent.

Other limitations included that capillary blood samples were analysed with point‐of‐care devices and may not accurately reflect venous circulation values by laboratory‐based measures, often by over‐estimating the R‐βHB concentration (Evans et al., 2022). Collection of blood gases would be necessary in order to confirm our hypothesized mechanism of action involving acidosis and declining pH, and unfortunately due to technical failures, the $S_{pO_{2}}$ results are in a subset (12 of 16, or 75%) of participants. Four participants were removed from the study due to not completing the period of hypoxic exposure, potentially introducing a bias into the results as these participants may have ultimately been non‐responders to the provided KME countermeasure. While we assessed normobaric hypoxia, hypobaric hypoxia may also result in physiological differences, which are not possible to assess or infer from in the present design (Coppel et al., 2015). Furthermore, due to the length and severity of the hypoxic exposure, it was deemed best practice to remove the ROBD prior to administering the final GI/AMS questionnaire, which may have affected the self‐reported hypoxia symptoms at that time point.

In conclusion, the decline in oxygen saturation during acute severe hypoxia was attenuated with KME compared to PLA, and coincided with an attenuation of decline in cognitive performance associated with acute rapid‐onset hypoxic exposure. Future work should investigate whether KME can attenuate declines in $S_{pO_{2}}$ and improve cognitive resilience in other contexts, given the potential for KME as a viable option to mitigate both environmental and pathological exposures to hypoxia (Shaw et al., 2021; Stubbs et al., 2020).

## AUTHOR CONTRIBUTIONS

Conceived and designed research: Jeffrey Phillips, Andrew P. Koutnik, Brianna J. Stubbs, Dawn Kernagis. Performed experiments: Tyler S. McClure, Jeffrey Phillips, Andrew P. Koutnik, Kody Coleman, Ed Chappe. Analysed data: Tyler S. McClure, Andrew P. Koutnik, Gary R. Cutter, Marcas M. Bamman. Interpreted results of experiments: Jeffrey Phillips, Tyler S. McClure, Andrew P. Koutnik, Gary R. Cutter, Marcas M. Bamman, Dawn Kernagis. Prepared figures: Tyler S. McClure, Gary R. Cutter, Brendan Egan, Marcas M. Bamman. Drafted manuscript: Tyler S. McClure, Brendan Egan, Marcas M. Bamman. Edited and revised manuscript: Tyler S. McClure, Jeffrey Phillips, Andrew P. Koutnik, Kody Coleman, Ed Chappe, Gary R. Cutter, Brendan Egan, Todd Norell, Brianna J. Stubbs, Marcas M. Bamman, Dawn Kernagis. Approved final version of manuscript: Tyler S. McClure, Jeffrey Phillips, Andrew P. Koutnik, Kody Coleman, Ed Chappe, Gary R. Cutter, Brendan Egan, Todd Norell, Brianna J. Stubbs, Marcas M. Bamman, Dawn Kernagis. All authors have read and approved the final version of this manuscript and agree to be accountable for all aspects of the work in ensuring that questions related to the accuracy or integrity of any part of the work are appropriately investigated and resolved. All persons designated as authors qualify for authorship, and all those who qualify for authorship are listed.

## CONFLICT OF INTEREST

H.V.M.N. Inc. had no involvement in data collection, analysis or data interpretation. Tyler S. McClure, Jeffrey Phillips, Kody Coleman, Ed Chappe, Dawn Kernagis, Brendan Egan, and Marcas M. Bamman declare no conflicts of interest, and do not have any financial disclosures. Brianna J. Stubbs has stock and stock options in companies that produce ketone products (H.V.M.N. Inc., Juvenescence Ltd, BHB Therapeutics Ltd, Selah Ltd) and is an inventor on patents that relate to ketone bodies. Brianna J. Stubbs was an employee of H.V.M.N. Inc. at the time this study was conceived. Gary R. Cutter participates on Data and Safety Monitoring Boards for Applied Therapeutics, AI therapeutics, AMO Pharma, Astra‐Zeneca, Avexis Pharmaceuticals, Bristol Meyers Squibb/Celgene, CSL Behring, Horizon Pharmaceuticals, Immunic, Karuna Therapeutics, Kezar Life Sciences, Mapi Pharmaceuticals LTD, Merck, Mitsubishi Tanabe Pharma Holdings, Opko Biologics, Prothena Biosciences, Novartis, Regeneron, Sanofi‐Aventis, Reata Pharmaceuticals, Teva Pharmaceuticals, NHLBI (Protocol Review Committee), University of Texas Southwestern, University of Pennsylvania, and Visioneering Technologies, Inc. Gary R. Cutter participates as a consultant or Advisory Board member for Alexion, Antisense Therapeutics, Avotres, Biogen, Clene Nanomedicine, Clinical Trial Solutions LLC, Entelexo Biotherapeutics, Inc., Genzyme, Genentech, GW Pharmaceuticals, Hoya Corporation, Immunic, Immunosis Pty Ltd, Klein‐Buendel Incorporated, Linical, Merck/Serono, Novartis, Perception Neurosciences, Protalix Biotherapeutics, Regeneron, Roche, and SAB Biotherapeutics. Gary R. Cutter is President of Pythagoras, Inc. a private consulting company located in Birmingham, AL, USA. Andrew P. Koutnik is an inventor on patents related to ketone bodies (US11452704B2; US11596616B2) and on the Scientific Advisory Board for Simply Good Foods and Nutri.

## Data Availability

The data that support the findings of this study are available upon request from the corresponding author.

## References

[eph13617-bib-0001] Asmaro, D. , Mayall, J. , & Ferguson, S. (2013). Cognition at altitude: Impairment in executive and memory processes under hypoxic conditions. Aviation, Space, and Environmental Medicine, 84(11), 1159–1165. 24279229 10.3357/asem.3661.2013

[eph13617-bib-0002] Beer, J. M. A. , Shender, B. S. , Chauvin, D. , Dart, T. S. , & Fischer, J. (2017). Cognitive deterioration in moderate and severe hypobaric hypoxia conditions. Aerospace Medicine and Human Performance, 88(7), 617–626. 28641678 10.3357/AMHP.4709.2017

[eph13617-bib-0003] Brownlow, M. L. , Jung, S. H. , Moore, R. J. , Bechmann, N. , & Jankord, R. (2017). Nutritional ketosis affects metabolism and behavior in sprague‐dawley rats in both control and chronic stress environments. Frontiers in Molecular Neuroscience, 10, 129. 28555095 10.3389/fnmol.2017.00129 PMC5430035

[eph13617-bib-0004] Burke, L. M. (2021). Ketogenic low‐CHO, high‐fat diet: The future of elite endurance sport? The Journal of Physiology, 599(3), 819–843. 32358802 10.1113/JP278928 PMC7891323

[eph13617-bib-0005] Chaillou, T. (2018). Skeletal muscle fiber type in hypoxia: Adaptation to high‐altitude exposure and under conditions of pathological hypoxia. Frontiers in Physiology, 9, 1450. https://www.frontiersin.org/article/10.3389/fphys.2018.01450 30369887 10.3389/fphys.2018.01450 PMC6194176

[eph13617-bib-0006] Coleman, K. , Phillips, J. , Sciarini, M. , Stubbs, B. , Jackson, O. , & Kernagis, D. (2021). A metabolic intervention for improving human cognitive performance during hypoxia. Aerospace Medicine and Human Performance, 92(7), 556–562. 34503629 10.3357/AMHP.5767.2021

[eph13617-bib-0007] Coppel, J. , Hennis, P. , Gilbert‐Kawai, E. , & Grocott, M. P. (2015). The physiological effects of hypobaric hypoxia versus normobaric hypoxia: A systematic review of crossover trials. Extreme Physiology & Medicine, 4, 2. 25722851 10.1186/s13728-014-0021-6 PMC4342204

[eph13617-bib-0008] Cunnane, S. C. , Trushina, E. , Morland, C. , Prigione, A. , Casadesus, G. , Andrews, Z. B. , Beal, M. F. , Bergersen, L. H. , Brinton, R. D. , de la Monte, S. , Eckert, A. , Harvey, J. , Jeggo, R. , Jhamandas, J. H. , Kann, O. , la Cour, C. M. , Martin, W. F. , Mithieux, G. , Moreira, P. I. , … Millan, M. J. (2020). Brain energy rescue: An emerging therapeutic concept for neurodegenerative disorders of ageing. Nature Reviews‐ Drug Discovery, 19(9), 609–633. 32709961 10.1038/s41573-020-0072-x PMC7948516

[eph13617-bib-0009] Dunham‐Snary, K. J. , Wu, D. , Sykes, E. A. , Thakrar, A. , Parlow, L. R. G. , Mewburn, J. D. , Parlow, J. L. , & Archer, S. L. (2017). Hypoxic pulmonary vasoconstriction: From molecular mechanisms to medicine. Chest, 151(1), 181–192. 27645688 10.1016/j.chest.2016.09.001 PMC5310129

[eph13617-bib-0010] Evans, M. , & Egan, B. (2018). Intermittent running and cognitive performance after ketone ester ingestion. Medicine & Science in Sports & Exercise, 50(11), 2330–2338. 29944604 10.1249/MSS.0000000000001700

[eph13617-bib-0011] Evans, M. , McClure, T. S. , Koutnik, A. P. , & Egan, B. (2022). Exogenous ketone supplements in athletic contexts: Past, present, and future. Sports Medicine, 52(Suppl1), 25–67. 10.1007/s40279-022-01756-2 PMC9734240 36214993

[eph13617-bib-0012] Gom, R. C. , Bhatt, D. , Villa, B. R. , George, A. G. , Lohman, A. W. , Mychasiuk, R. , Rho, J. M. , & Teskey, G. C. (2021). The ketogenic diet raises brain oxygen levels, attenuates postictal hypoxia, and protects against learning impairments. Neurobiology of Disease, 154, 105335. 33741453 10.1016/j.nbd.2021.105335

[eph13617-bib-0013] Goodall, S. , Twomey, R. , & Amann, M. (2014). Acute and chronic hypoxia: Implications for cerebral function and exercise tolerance. Fatigue: Biomedicine, Health & Behavior, 2(2), 73–92. 10.1080/21641846.2014.909963 PMC4292893 25593787

[eph13617-bib-0014] Huang, R. , Huestis, M. , Gan, E. S. , Ooi, E. E. , & Ohh, M. (2021). Hypoxia and viral infectious diseases. Journal of Clinical Investigation Insight, 6(7), e147190. 33830079 10.1172/jci.insight.147190 PMC8119216

[eph13617-bib-0015] Karamanli, H. , Ilik, F. , Kayhan, F. , & Pazarli, A. C. (2015). Assessment of cognitive impairment in long‐term oxygen therapy‐dependent COPD patients. International Journal of Chronic Obstructive Pulmonary Disease, 10, 2087–2094. 26491279 10.2147/COPD.S88326 PMC4598205

[eph13617-bib-0016] Kushida, C. A. (2004). Sleep deprivation: Clinical issues, pharmacology, and sleep loss effects. CRC Press.

[eph13617-bib-0017] Lathan, C. , Spira, J. L. , Bleiberg, J. , Vice, J. , & Tsao, J. W. (2013). Defense Automated Neurobehavioral Assessment (DANA)‐psychometric properties of a new field‐deployable neurocognitive assessment tool. Military Medicine, 178(4), 365–371. 23707818 10.7205/MILMED-D-12-00438

[eph13617-bib-0018] Leaf, D. E. , & Goldfarb, D. S. (2007). Mechanisms of action of acetazolamide in the prophylaxis and treatment of acute mountain sickness. Journal of Applied Physiology, 102(4), 1313–1322. 17023566 10.1152/japplphysiol.01572.2005

[eph13617-bib-0019] Legg, S. J. , Gilbey, A. , Hill, S. , Raman, A. , Dubray, A. , Iremonger, G. , & Mündel, T. (2016). Effects of mild hypoxia in aviation on mood and complex cognition. Applied Ergonomics, 53 Pt B, 357–363. 26482893 10.1016/j.apergo.2015.10.002

[eph13617-bib-0020] Malle, C. , Quinette, P. , Laisney, M. , Bourrilhon, C. , Boissin, J. , Desgranges, B. , Eustache, F. , & Piérard, C. (2013). Working memory impairment in pilots exposed to acute hypobaric hypoxia. Aviation, Space, and Environmental Medicine, 84(8), 773–779. 23926651 10.3357/asem.3482.2013

[eph13617-bib-0021] Mank, M. M. , Reed, L. F. , Walton, C. J. , Barup, M. L. T. , Ather, J. L. , & Poynter, M. E. (2022). Therapeutic ketosis decreases methacholine hyperresponsiveness in mouse models of inherent obese asthma. American Journal of Physiology‐ Lung Cellular and Molecular Physiology, 322(2), L243–L257. 34936508 10.1152/ajplung.00309.2021 PMC8782644

[eph13617-bib-0022] Michiels, C. (2004). Physiological and pathological responses to hypoxia. The American Journal of Pathology, 164(6), 1875–1882. 15161623 10.1016/S0002-9440(10)63747-9 PMC1615763

[eph13617-bib-0023] Mujica‐Parodi, L. R. , Amgalan, A. , Sultan, S. F. , Antal, B. , Sun, X. , Skiena, S. , Lithen, A. , Adra, N. , Ratai, E.‐M. , Weistuch, C. , Govindarajan, S. T. , Strey, H. H. , Dill, K. A. , Stufflebeam, S. M. , Veech, R. L. , & Clarke, K. (2020). Diet modulates brain network stability, a biomarker for brain aging, in young adults. Proceedings of the National Academy of Sciences, USA, 117(11), 6170–6177. 10.1073/pnas.1913042117 PMC7084077 32127481

[eph13617-bib-0024] Murray, A. J. , Knight, N. S. , Cole, M. A. , Cochlin, L. E. , Carter, E. , Tchabanenko, K. , Pichulik, T. , Gulston, M. K. , Atherton, H. J. , Schroeder, M. A. , Deacon, R. M. J. , Kashiwaya, Y. , King, M. T. , Pawlosky, R. , Rawlins, J. N. P. , Tyler, D. J. , Griffin, J. L. , Robertson, J. , Veech, R. L. , & Clarke, K. (2016). Novel ketone diet enhances physical and cognitive performance. The Federation of American Societies for Experimental Biology Journal, 30(12), 4021–4032. 27528626 10.1096/fj.201600773R PMC5102124

[eph13617-bib-0025] Norwitz, N. G. , Winwood, R. , Stubbs, B. J. , D'Agostino, D. P. , & Barnes, P. J. (2021). Case report: Ketogenic diet is associated with improvements in chronic obstructive pulmonary disease. Frontiers in Medicine, 8, 699427. 34395478 10.3389/fmed.2021.699427 PMC8358145

[eph13617-bib-0026] Ochi, G. , Kanazawa, Y. , Hyodo, K. , Suwabe, K. , Shimizu, T. , Fukuie, T. , Byun, K. , & Soya, H. (2018). Hypoxia‐induced lowered executive function depends on arterial oxygen desaturation. The Journal of Physiological Sciences, 68(6), 6. 10.1007/s12576-018-0603-y PMC10717617 29536370

[eph13617-bib-0027] Pasiakos, S. M. , Karl, J. P. , & Margolis, L. M. (2021). Challenging traditional carbohydrate intake recommendations for optimizing performance at high altitude. Current Opinion in Clinical Nutrition & Metabolic Care, 24(6), 483–489. 34284412 10.1097/MCO.0000000000000782

[eph13617-bib-0028] Pavlov, N. , Haynes, A. G. , Stucki, A. , Jüni, P. , & Ott, S. R. (2018). Long‐term oxygen therapy in COPD patients: Population‐based cohort study on mortality. International Journal of Chronic Obstructive Pulmonary Disease, 13, 979–988. 29606865 10.2147/COPD.S154749 PMC5868621

[eph13617-bib-0029] Poff, A. M. , Koutnik, A. P. , & Egan, B. (2020). Nutritional ketosis with ketogenic diets or exogenous ketones: Features, convergence, and divergence. Current Sports Medicine Reports, 19(7), 251–259. 32692060 10.1249/JSR.0000000000000732

[eph13617-bib-0030] Poff, A. M. , Moss, S. , Soliven, M. , & D'Agostino, D. P. (2021). Ketone supplementation: Meeting the needs of the brain in an energy crisis. Frontiers in Nutrition, 8, 783659. 35004814 10.3389/fnut.2021.783659 PMC8734638

[eph13617-bib-0031] Poffe, C. , Robberechts, R. , Podlogar, T. , Kusters, M. , Debevec, T. , & Hespel, P. (2021). Exogenous ketosis increases blood and muscle oxygenation but not performance during exercise in hypoxia. American Journal of Physiology‐ Regulatory, Integrative and Comparative Physiology, 321(6), R844–R857. 34668436 10.1152/ajpregu.00198.2021

[eph13617-bib-0032] Poffé, C. , Robberechts, R. , Stalmans, M. , Vanderroost, J. , Bogaerts, S. , & Hespel, P. (2023). Exogenous ketosis increases circulating dopamine concentration and maintains mental alertness in ultra‐endurance exercise. Journal of Applied Physiology, 134(6), 1456–1469. 37141424 10.1152/japplphysiol.00791.2022

[eph13617-bib-0033] Posch, A. M. , Dandorf, S. , & Hile, D. C. (2018). The effects of acetazolamide on exercise performance at sea level and in hypoxic environments: A review. Wilderness & Environmental Medicine, 29(4), 541–545. 30314664 10.1016/j.wem.2018.06.011

[eph13617-bib-0034] Prins, P. J. , Buxton, J. D. , McClure, T. S. , D'Agostino, D. P. , Ault, D. L. , Welton, G. L. , Jones, D. W. , Atwell, A. D. , Slack, M. A. , Slack, M. L. , Williams, C. E. , Blanchflower, M. E. , Kannel, K. K. , Faulkner, M. N. , Szmaciasz, H. L. , Croll, S. M. , Stanforth, L. M. , Harris, T. D. , Gwaltney, H. C. , & Koutnik, A. P. (2021). Ketone bodies impact on hypoxic CO2 retention protocol during exercise. Frontiers in Physiology, 12, 780755. 34966291 10.3389/fphys.2021.780755 PMC8711099

[eph13617-bib-0035] Prins, P. J. , D'Agostino, D. P. , Rogers, C. Q. , Ault, D. L. , Welton, G. L. , Jones, D. W. , Henson, S. R. , Rothfuss, T. J. , Aiken, K. G. , Hose, J. L. , England, E. L. , Atwell, A. D. , Buxton, J. D. , & Koutnik, A. P. (2020). Dose response of a novel exogenous ketone supplement on physiological, perceptual and performance parameters. Nutrition & Metabolism, 17, 81. 33005207 10.1186/s12986-020-00497-1 PMC7523040

[eph13617-bib-0036] Robach, P. , Calbet, J. A. L. , Thomsen, J. J. , Boushel, R. , Mollard, P. , Rasmussen, P. , & Lundby, C. (2008). The ergogenic effect of recombinant human erythropoietin on VO2max depends on the severity of arterial hypoxemia. PLoS ONE, 3(8), e2996. 18714372 10.1371/journal.pone.0002996 PMC2500186

[eph13617-bib-0037] Roberts, C. M. , Murray, N. , Hunfalvay, M. , & Lange, B. (2017). Reliability and normative data of computerized dynamic visual acuity tests. Otology & Neurotology, 37(5), 545–552. 10.1097/MAO.0000000000001014 27002314

[eph13617-bib-0038] Ryu, S. , Shchukina, I. , Youm, Y.‐H. , Qing, H. , Hilliard, B. , Dlugos, T. , Zhang, X. , Yasumoto, Y. , Booth, C. J. , Fernández‐Hernando, C. , Suárez, Y. , Khanna, K. , Horvath, T. L. , Dietrich, M. O. , Artyomov, M. , Wang, A. , & Dixit, V. D. (2021). Ketogenic diet restrains aging‐induced exacerbation of coronavirus infection in mice. eLife, 10, e66522. 34151773 10.7554/eLife.66522 PMC8245129

[eph13617-bib-0039] Sarkar, M. , Niranjan, N. , & Banyal, P. (2017). Mechanisms of hypoxemia. Lung India, 34(1), 47–60. 28144061 10.4103/0970-2113.197116 PMC5234199

[eph13617-bib-0040] Shaw, D. M. , Cabre, G. , & Gant, N. (2021). Hypoxic hypoxia and brain function in military aviation: Basic physiology and applied perspectives. Frontiers in Physiology, 12, 665821. https://www.frontiersin.org/article/10.3389/fphys.2021.665821 34093227 10.3389/fphys.2021.665821 PMC8171399

[eph13617-bib-0041] Snetselaar, L. G. , de Jesus, J. M. , DeSilva, D. M. , & Stoody, E. E. (2021). Dietary guidelines for Americans, 2020–2025. Nutrition Today, 56(6), 287–295. 34987271 10.1097/NT.0000000000000512 PMC8713704

[eph13617-bib-0042] Stepanek, J. , Pradhan, G. N. , Cocco, D. , Smith, B. E. , Bartlett, J. , Studer, M. , Kuhn, F. , & Cevette, M. J. (2014). Acute hypoxic hypoxia and isocapnic hypoxia effects on oculometric features. Aviation, Space, and Environmental Medicine, 85(7), 700–707. 25022157 10.3357/asem.3645.2014

[eph13617-bib-0043] Stubbs, B. J. , Koutnik, A. P. , Goldberg, E. L. , Upadhyay, V. , Turnbaugh, P. J. , Verdin, E. , & Newman, J. C. (2020). Investigating ketone bodies as immunometabolic countermeasures against respiratory viral infections. Medicine, 1(1), 43–65. 10.1016/j.medj.2020.06.008 PMC7362813 32838361

[eph13617-bib-0044] Subar, A. F. , Kirkpatrick, S. I. , Mittl, B. , Zimmerman, T. P. , Thompson, F. E. , Bingley, C. , Willis, G. , Islam, N. G. , Baranowski, T. , McNutt, S. , & Potischman, N. (2012). The Automated Self‐Administered 24‐Hour Dietary Recall (ASA24): A resource for researchers, clinicians and educators from the national cancer institute. Journal of the Academy of Nutrition and Dietetics, 112(8), 1134–1137. 22704899 10.1016/j.jand.2012.04.016 PMC3721511

[eph13617-bib-0045] Subudhi, A. W. , Dimmen, A. C. , & Roach, R. C. (2007). Effects of acute hypoxia on cerebral and muscle oxygenation during incremental exercise. Journal of Applied Physiology, 103(1), 177–183. 17431082 10.1152/japplphysiol.01460.2006

[eph13617-bib-0046] Sun, M.‐K. , & Alkon, D. L. (2002). Carbonic anhydrase gating of attention: Memory therapy and enhancement. Trends in Pharmacological Sciences, 23(2), 83–89. 11830265 10.1016/s0165-6147(02)01899-0

[eph13617-bib-0047] Turner, C. E. , Barker‐Collo, S. L. , Connell, C. J. W. , & Gant, N. (2015). Acute hypoxic gas breathing severely impairs cognition and task learning in humans. Physiology & Behavior, 142, 104–110. 25660759 10.1016/j.physbeh.2015.02.006

[eph13617-bib-0048] Volek, J. S. , Yancy, W. S. , Gower, B. A. , Phinney, S. D. , Slavin, J. , Koutnik, A. P. , Hurn, M. , Spinner, J. , Cucuzzella, M. , & Hecht, F. M. (2024). Expert consensus on nutrition and lower‐carbohydrate diets: An evidence‐ and equity‐based approach to dietary guidance. Frontiers in Nutrition, 11, 1376098. 38487629 10.3389/fnut.2024.1376098 PMC10937533

[eph13617-bib-0049] Waldman, H. S. , Basham, S. A. , Price, F. G. , Smith, J. W. , Chander, H. , Knight, A. C. , Krings, B. M. , & McAllister, M. J. (2018). Exogenous ketone salts do not improve cognitive responses after a high‐intensity exercise protocol in healthy college‐aged males. Applied Physiology, Nutrition, and Metabolism, 43(7), 711–717. 10.1139/apnm-2017-0724 29451991

[eph13617-bib-0050] Walsh, J. J. , Caldwell, H. G. , Neudorf, H. , Ainslie, P. N. , & Little, J. P. (2021). Short‐term ketone monoester supplementation improves cerebral blood flow and cognition in obesity: A randomized cross‐over trial. The Journal of Physiology, 599(21), 4763–4778. 34605026 10.1113/JP281988

[eph13617-bib-0051] Walsh, J. J. , Myette‐Côté, É. , & Little, J. P. (2020). The effect of exogenous ketone monoester ingestion on plasma BDNF during an oral glucose tolerance test. Frontiers in Physiology, 11, 1094. 33013465 10.3389/fphys.2020.01094 PMC7509175

[eph13617-bib-0052] Wang, J. , Ke, T. , Zhang, X. , Chen, Y. , Liu, M. , Chen, J. , & Luo, W. (2013). Effects of acetazolamide on cognitive performance during high‐altitude exposure. Neurotoxicology and Teratology, 35, 28–33. 23280141 10.1016/j.ntt.2012.12.003

[eph13617-bib-0053] Wang, K. , Smith, Z. M. , Buxton, R. B. , Swenson, E. R. , & Dubowitz, D. J. (2015). Acetazolamide during acute hypoxia improves tissue oxygenation in the human brain. Journal of Applied Physiology, 119(12), 1494–1500. 26472861 10.1152/japplphysiol.00117.2015 PMC4683345

[eph13617-bib-0054] Williams, T. B. , Corbett, J. , McMorris, T. , Young, J. S. , Dicks, M. , Ando, S. , Thelwell, R. C. , Tipton, M. J. , & Costello, J. T. (2019). Cognitive performance is associated with cerebral oxygenation and peripheral oxygen saturation, but not plasma catecholamines, during graded normobaric hypoxia. Experimental Physiology, 104(9), 1384–1397. 31192502 10.1113/EP087647

